# Fabrication and Performance of Thermally Triggered Active Fire-Extinguishing Patches Based on Perfluorohexanone Microcapsule-Modified Epoxy Resin

**DOI:** 10.3390/polym18212640

**Published:** 2026-10-25

**Authors:** Zihou Tian, Le Yang, Songdi Zhang, Xiaoqian Wang, Xinteng Li, Jinliang An

**Affiliations:** 1 School of Civil Engineering, Hebei University of Engineering, Handan 056000, China; 2 Department of Mechanical and Aerospace Engineering, The Hong Kong University of Science and Technology, Clear Water Bay, Kowloon, Hong Kong, China; 3 School of Economics and Management, Yanshan University, Qinghuangdao 066004, China

**Keywords:** perfluorohexanone, microencapsulation, thermally triggered release, epoxy resin, active fire-extinguishing patch

## Abstract

To address the challenges of timely detection and local suppression of incipient fires, as well as the inherent limitations of traditional flame-retardant materials that primarily rely on passive thermal insulation or combustion retardation, this study developed thermally responsive microcapsules with perfluorohexanone as the core material and melamine–urea–formaldehyde (MUF) resin as the shell. These microcapsules were incorporated into an epoxy resin matrix to fabricate active fire-extinguishing patches. The morphology, chemical structure, thermal stability, thermally triggered release behavior, and fire-extinguishing performance of the microcapsules and the composite patches were systematically characterized by microscopic observation, FTIR, XPS, TGA particle size analysis, and combustion tests. The results showed that the prepared microcapsules exhibited a nearly spherical morphology with a median particle size (D_50_) of 143.94 μm and an encapsulation efficiency of 87.78%. After heating at 60 °C for 24 h, the mass loss was 5.88%, and after sealed storage at room temperature for 360 days, the mass loss was only 1.8%, indicating satisfactory thermal stability and storage stability. When the microcapsules were compounded with epoxy resin, the self-extinguishing time of the resulting patches decreased with increasing microcapsule content, reaching a minimum of 4.81 s. Mechanism analysis suggested that the fire-extinguishing effect of the composite system originated predominantly from gas-phase flame inhibition by the released perfluorohexanone, possibly synergized with a condensed-phase barrier effect derived from the charring epoxy matrix upon heating. This work provides experimental evidence for the application of perfluorohexanone-based active fire-extinguishing composites in proactive fire protection.

## 1. Introduction

According to global fire data released by the World Fire Statistics Center, fires cause approximately 20,600 deaths and 69,500 injuries worldwide each year, with residential building fires accounting for a high proportion [1,2,3]. The escalation of many fire incidents is closely related to the failure to promptly detect and effectively control incipient fire sources. Therefore, for high-risk locations such as combustible substrates, energy storage batteries, electrical equipment, and confined spaces, developing functional materials that combine flame-retardant protection with thermally triggered fire-extinguishing capabilities is of great significance for delaying ignition, suppressing flame spread, and extinguishing incipient fire sources.

Halon fire-extinguishing agents are being progressively restricted and phased out under the Montreal Protocol and its subsequent amendments due to their ozone-depleting effect [4]. Perfluorohexanone (C_6_F_12_O, commonly known as FK-5-1-12 in engineering; hereafter abbreviated as PHF in this work) features a zero-ozone depletion potential (ODP = 0), a low global warming potential (GWP ≈ 1), good electrical insulation, and no solid residue after extinguishment, making it a promising candidate to replace halon-based agents [5,6,7,8,9,10,11]. However, PHF has a boiling point of approximately 49 °C at atmospheric pressure and a relatively high saturated vapor pressure, rendering it prone to volatilization at ambient temperature and difficult to stably retain in solid media such as flexible substrates, coatings, or resins over the long term. Its compatibility with most polymer matrices is also limited, and direct blending tends to cause migration or leakage during processing, curing, and service, thereby compromising long-term fire-extinguishing performance [12,13].

Microencapsulated fire-extinguishing agents represent a category of responsive materials that have advanced rapidly in the field of fire protection functional materials in recent years. By virtue of a core–shell structural design, liquid or solid fire-extinguishing components can be encapsulated within a shell and released upon temperature rise, shell softening, rupture, or increased permeability, thereby enabling local fire suppression or flame retardancy [14,15,16,17,18,19,20,21]. Previous studies have shown that stearate-coated expandable graphite particles can be employed for sodium fire suppression [22]; microcapsules with an octadecane/tributyl phosphate hybrid core and a silica or poly(urea–formaldehyde) shell can exhibit distinct thermal stability and flame-retardant effects [23]; gel-type core–shell structured powders can synergistically inhibit flames through combined physical and chemical mechanisms [24]; and temperature-responsive polymer-shell self-extinguishing microcapsules can provide a degree of self-extinguishing protection in lithium battery systems [25]. These studies indicate that microencapsulation is an effective strategy for addressing the challenges associated with the storage, dispersion, and thermally triggered release of fire-extinguishing agents. Nevertheless, the stable encapsulation and controlled release of volatile perfluorohexanone within a resin matrix still require further investigation.

Epoxy resins are an important class of thermosetting polymers that possess good adhesion, electrical insulation, mechanical properties, and chemical resistance, and are extensively used in fields such as new energy, electronics and electrical systems, structural bonding, and protective coatings [26,27,28]. However, conventional epoxy resins are flammable, exhibit a low limiting oxygen index (LOI), and may generate smoke and pyrolysis products during combustion. Traditional flame-retardant modifications generally focus on reducing flammability or promoting char formation, but are incapable of actively extinguishing incipient flames [29,30,31,32,33,34]. Therefore, the introduction of thermally responsive perfluorohexanone microcapsules into an epoxy resin is expected to impart the material with a local thermally triggered fire-extinguishing function while preserving the film-forming and adhesion capabilities of the matrix, thereby expanding the application scope of epoxy-based composites in proactive fire protection.

In this study, perfluorohexanone microcapsules (PHF MCs) were prepared via in situ polymerization, and their chemical composition, micromorphology, thermal stability, and release behavior were characterized. On this basis, PHF MCs were incorporated into an epoxy resin matrix and cured to fabricate thermally triggered active fire-extinguishing patches, and the combustion self-extinguishing behavior and basic surface/mechanical properties of the patches were evaluated. This work focuses on verifying the dispersibility, storage stability, and thermally triggered fire-extinguishing effectiveness of perfluorohexanone microcapsules in epoxy resin, while discussing the possible synergistic mechanism of gas-phase flame inhibition and condensed-phase barrier effects.

## 2. Materials and Methods

### 2.1. Materials

All reagents are commercial products and have not been further purified before use. Perfluorohexanone (PHF)(C_6_F_12_O, AR, ≥99.7%, Beijing Yuji Technology Development Co., Ltd., Beijing, China) was used as the core material. Melamine (C_3_H_6_N_6_, Shanghai Aladdin Biochemical Technology Co., Ltd., Shanghai, China), formaldehyde solution (CH_2_O, 37 wt% aqueous solution, containing 10–15% methanol stabilizer, Shanghai Aladdin Biochemical Technology Co., Ltd., Shanghai, China) and urea (CH_4_N_2_O, Shanghai Aladdin Biochemical Technology Co., Ltd., Shanghai, China) were used as the MUF shell precursors. Poly(vinyl alcohol) (PVA, (C_2_H_4_O)_n_, Shanghai Aladdin Biochemical Technology Co., Ltd., Shanghai, China) and poly(ethylene-co-maleic anhydride) (EMA, analytical grade, Hubei Baidu Chemical Co., Ltd., Tianmen, China) were employed as emulsifying/dispersing stabilizers. Resorcinol (C_6_H_6_O_2_, industrial grade, AR, ≥99.7%, Zhejiang Hongsheng Chemical Co., Ltd., Zhejiang, China) served as a shell modification or crosslinking auxiliary component. Sodium chloride (NaCl, analytical grade, Xilong Scientific Co., Ltd., Guangzhou, China) was used to adjust the ionic strength of the continuous phase. Dilute sulfuric acid (H_2_SO_4_, concentration 1 mol/L, Sinopharm Chemical Reagent Co., Ltd., Shanghai, China) was applied for pH adjustment. Epoxy resin (E-51, epoxy equivalent weight 184–195 g/eq, density about 1.17 g/cm^3^ at 25 °C, and viscosity 10–15 pa·s at 25 °C, Guangzhou Yuanyi Chemical Co., Ltd., Guangzhou, China) and T-31 epoxy curing agent (Shanghai Macklin Biochemical Co., Ltd., Shanghai, China) were used to prepare the composite patches.

### 2.2. Preparation of PHF MCs

(1) Synthesis of MUF Prepolymer

First, 62 g of formaldehyde solution, 20.835 g of urea, and 30 g of deionized water were added to a beaker and stirred until completely dissolved. Subsequently, 5.7 g of melamine was introduced, and the pH of the system was adjusted to 8.7. The mixture was heated from room temperature to 72 °C and stirred in an oil bath for 40 min to obtain the MUF prepolymer solution.

(2) Preparation of Core Material Emulsion

Masses of 15 g of PVA, 15 g of EMA, and 270 g of deionized water were added to a beaker, heated to 80 °C, and stirred for 60 min to fully dissolve the dispersing stabilizers, followed by cooling to room temperature. Subsequently, 0.7 g of NaCl, 3 g of resorcinol, and 100 g of PHF were introduced, and a PHF emulsion was obtained via homogenization.

(3) In Situ Polymerization and Preparation of Perfluorohexanone Microcapsules

**Figure 1:**
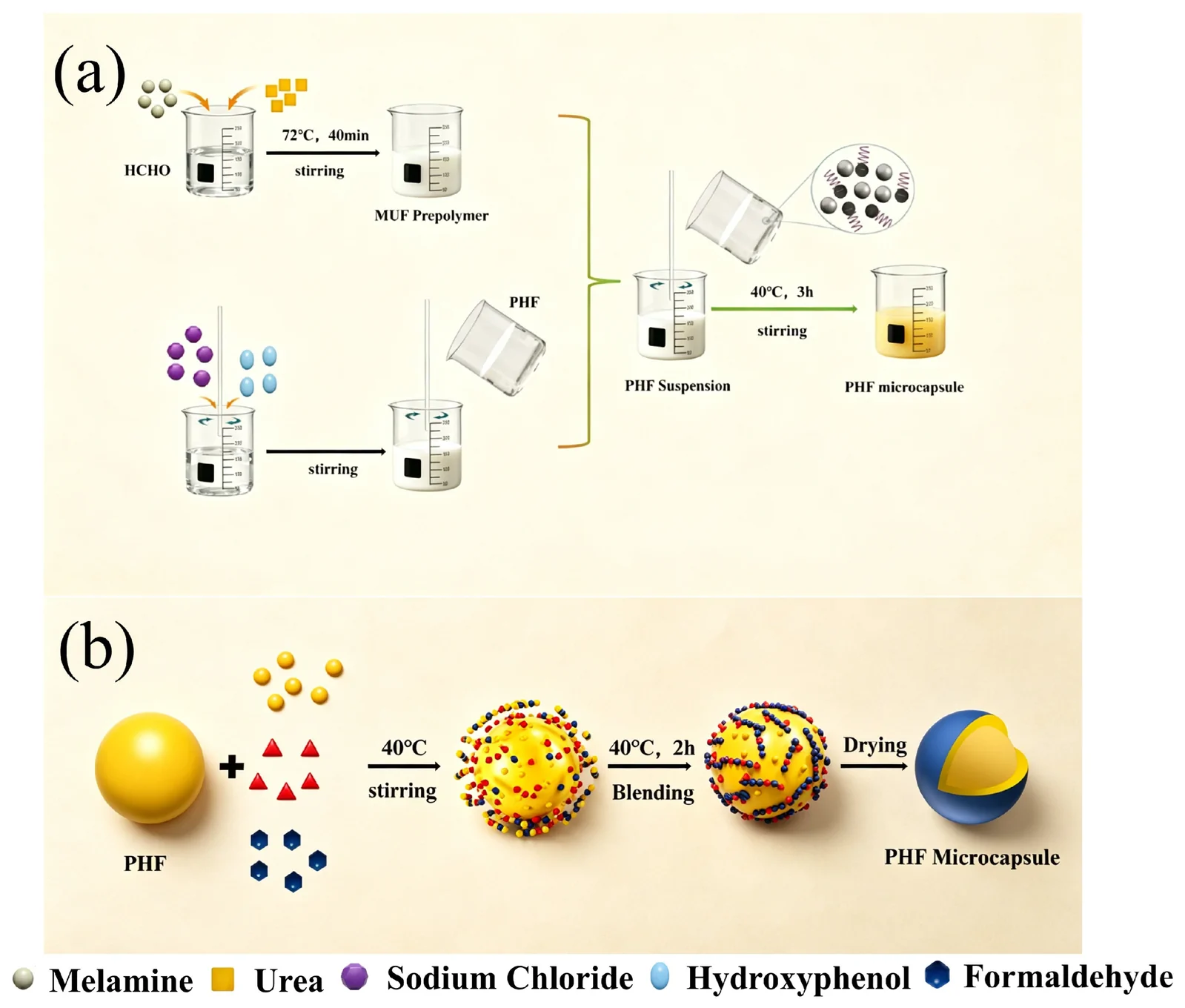
Fabrication process of PHF microcapsules: (**a**) schematic flow chart of synthesis; (**b**) schematic diagram of formation mechanism.

The MUF prepolymer solution was slowly added dropwise to the PHF emulsion, and the pH of the system was adjusted to 3.0 using 1 mol/L dilute sulfuric acid to induce the deposition and solidification of the MUF shell at the droplet interface. The system was further stirred for 3 h to obtain a microcapsule suspension. After standing for 1 h, the precipitate was collected and thoroughly washed with deionized water. The resulting microcapsules were dried at 30 °C for 12 h and designated as PHF MCs (Figure 1).

### 2.3. Fabrication of Epoxy-Based Fire Suppression Composites

PHF MCs with different mass fractions were mixed with epoxy resin according to the formulations designed in Table 1. The mixtures were first subjected to low-speed mechanical stirring and then underwent a short dispersion treatment under conditions that avoided microcapsule rupture and any pronounced temperature rise. Subsequently, the T-31 curing agent was added at a curing agent/epoxy resin mass ratio of 20/80, and the mixture was thoroughly blended and vacuum-defoamed. The obtained slurry was poured into a mold paved with a release film in advance, and the slurry was evenly spread and the thickness of the sheet was controlled by die pressing. After curing at room temperature for 24 h, the composite patch with flat surface was obtained by demoulding and peeling off the release film.

### 2.4. Characterization Techniques

Optical microscopy: The surface morphology, sphericity, particle size uniformity, and damage of the microcapsules were observed using an XSP-2CA binocular optical microscope.

Scanning electron microscopy (SEM) and energy-dispersive X-ray spectroscopy (EDS): The morphology of the samples was examined using a Hitachi SU-8220 field-emission scanning electron microscope at an accelerating voltage of 5 kV. Elemental distribution was analyzed by EDS.

Contact angle measurement: Using a Dataphysics OCA50 automatic contact angle measuring instrument, the contact angle was measured by the θ/2 method using ultrapure water as the test liquid. A droplet of approximately 1 μL was placed on the sample surface, and the image was acquired to calculate the contact angle. The test surface of the sample was the side close to the release film during compression molding. After demoulding and peeling off the release film, the surface was smooth and flat without extra grinding or polishing. Each sample was measured at least three times, and the average value was used to evaluate changes in the surface wettability of the composite.

Fourier transform infrared spectroscopy (FTIR) test: A Thermo Nicolet iS50 Fourier transform infrared spectrometer (Thermo Fisher Scientific, Waltham, MA, USA) was used, with the wave number range of 400–4000 cm^−1^ and the resolution of 4 cm, and the cumulative scanning was 32 times. The transmission spectrum of microcapsule powder was collected after KBr tableting; The surface spectrum of the composite patch was directly measured by diamond attenuated total reflection (ATR) attachment. In order to confirm the consistency of chemical structure under different filling amounts, 50% PHF MCs was selected for ATR-FTIR characterization.

X-ray photoelectron spectroscopy (XPS): XPS measurements were performed on a Shimadzu AXIS SUPRA+ spectrometer using monochromatic Al Kα X-rays (photon energy = 1486.6 eV) as the excitation source.

TGA test: Samples placed in alumina crucibles were heated to 600 °C at 10 °C/min under nitrogen atmosphere using a PerkinElmer TGA8000 SP3 Clarus-SQ8 instrument.

Particle size distribution: A Mastersizer 3000 (Malvern Panalytical, Malvern, UK) was used, and deionized water was used as the dispersion medium. After ultrasonic dispersion, the samples were added into the sample pool, and the light shielding rate was 10–20%. The average particle size distribution of microcapsules was measured by a Mastersizer 3000 laser particle size analyzer.

Shore hardness test: Shore A hardness (unit HA) of the composite patch was measured by Shore A rubber hardness tester. The thickness of the sample was not less than 3 mm, the indenter was vertically pressed on the flat surface, and the reading was kept for 15 s; each sample was measured 5 times at different positions and averaged.

Storage stability and mass loss test: The sample mass was weighed by electronic analytical balance (accuracy 0.1 mg). According to the thermal stability test of microcapsules, about 5.0 g powder was put into an open weighing bottle, kept at a constant temperature of 24 h in a 60 °C air drying oven, cooled to room temperature in a dryer and weighed. The mass loss rate was (*m*_0_ − *m*_1_)/*m*_0_ × 100%.

Long-term storage stability test: The microcapsules were sealed in aluminum foil bags, placed in a dark environment at room temperature (25 ± 2 °C), taken out and weighed at set time intervals. The composite materials were weighed before and after the butane flame extinguishing test, and the mass loss rate was (*m_a_* − *m_b_*)/*m_a_* × 100%.

Fire suppression test: A butane flame served as the ignition source. Vertically placed samples were exposed to the flame with a fixed vertical distance of 8 cm. The ignition self-extinguishing time was recorded.

UL-94 vertical combustion test: The sample size was 125 mm × 13 mm × 3 mm, and each group had at least 5 parallel samples. Before testing, the sample was pretreated for 48 h at 23 ± 2 °C and 50 ± 5% relative humidity. During the test, the sample was vertically fixed on the bracket, the lower end of the sample was 10 mm away from the Bunsen burner lamp mouth, the flame height was adjusted to 20 mm, and blue flame was adopted. The fire source was removed 10 s after the first flame application, and the residual flame time t_1_ was recorded. Immediately after the afterflame was extinguished, the second flame was applied for 10 s, and the afterflame time t_2_ was recorded. At the same time, it was observed and recorded whether the molten droplets ignited the absorbent cotton below.

## 3. Results and Discussion

### 3.1. Morphology Analysis of PHF MCs

**Figure 2:**
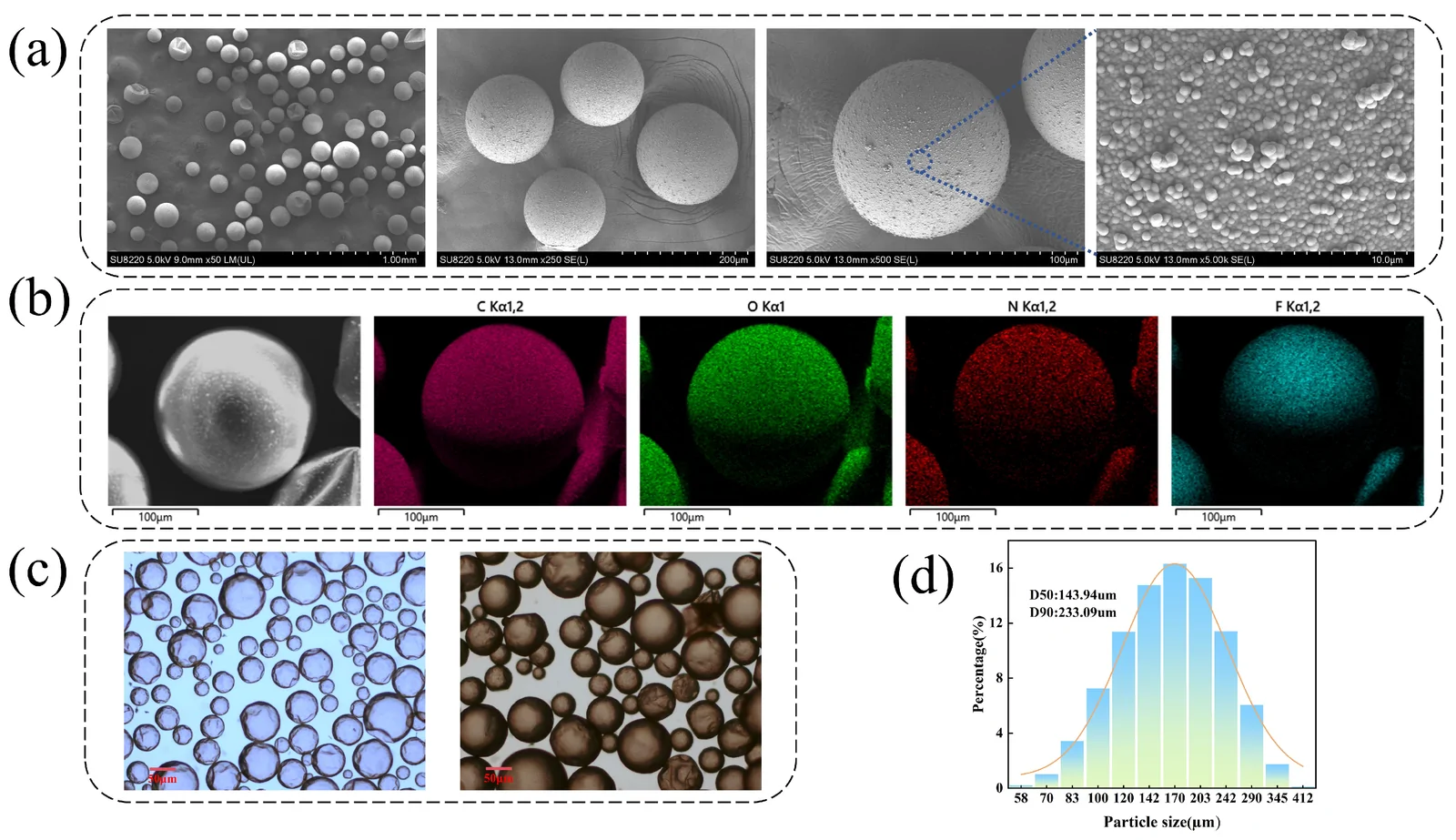
(**a**) SEM image of PHF MCs; (**b**) EDS elemental mapping; (**c**) optical microscopy images of PHF MCs after preparation and after drying; (**d**) particle size distribution of PHF MCs.

To obtain perfluorohexanone microcapsules capable of thermally triggered release, in situ polymerization was employed in this work to deposit a melamine–urea–formaldehyde (MUF) shell on the surface of PHF droplets, thereby enhancing the storage stability of the volatile core material. The underlying design principle is as follows: at ambient temperature, the MUF shell suppresses the volatilization of PHF, while upon heating, the internal pressure generated by vaporization of the core, together with shell softening or localized rupture, enables the release of PHF. Optical microscopy and SEM were used to observe the morphology of the microcapsules. As shown in Figure 2a, the resulting microcapsules were generally near-spherical with good dispersibility and without large-scale agglomeration. The high-magnification SEM image revealed a granular rough texture on the surface, which may be associated with the gradual deposition of the MUF prepolymer at the interface. Surface smoothness, sphericity and particle size uniformity are higher than those of microcapsules prepared by other methods [8,14]. EDS elemental mapping detected C, O, N, and F, among which N originated mainly from the MUF shell and F from the fluorinated core or its residue, providing direct chemical evidence for the core–shell structure and confirming the successful encapsulation of perfluorohexanone. Optical microscopy showed that the samples maintained a relatively regular particulate morphology in both wet and dry states. Particle size distribution results indicated that the PHF MCs had a median particle size (D_50_) of 143.94 μm with a relatively narrow distribution.

### 3.2. Chemical Composition and Storage Stability Analysis of PHF MCs

**Figure 3:**
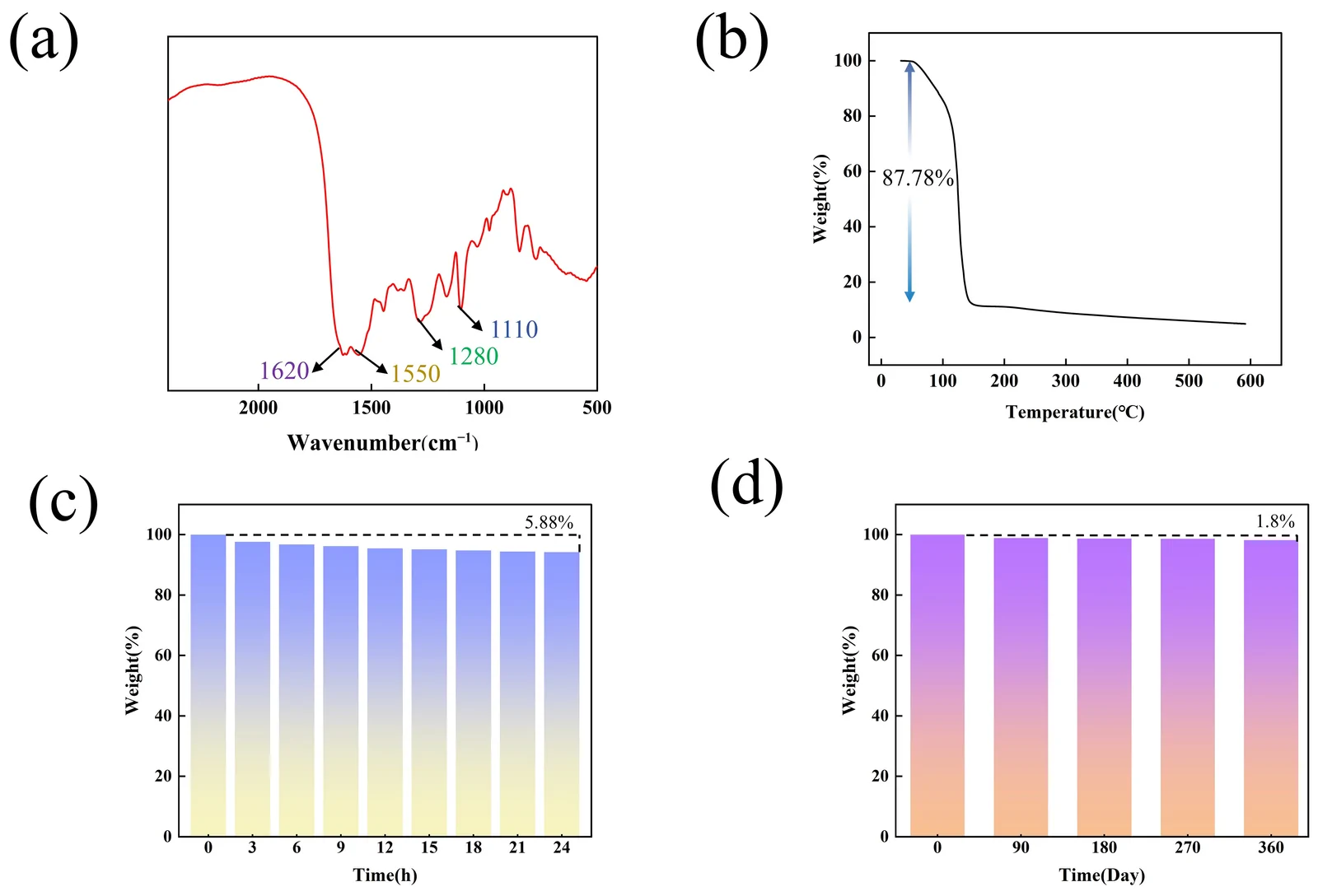
(**a**) FTIR spectra of PHF MCs; (**b**) TGA curve of PHF MCs; (**c**) mass loss of PHF MCs after heating at 60 °C for 24 h; (**d**) storage stability of PHF MCs within 360 days.

Figure 3 presents the chemical structure, thermal mass loss behavior, and storage stability of the PHF MCs. Figure 3a shows the FTIR spectrum of the PHF MCs. The peak near 1620 cm^−1^ can be assigned to C=O or amide-related vibrations in the MUF shell, and the peak near 1550 cm^−1^ can be attributed to C=N vibrations of the triazine ring, indicating the formation of the MUF shell. The strong C–F stretching vibration peaks observed in the range of 1100–1280 cm^−1^ indicate the presence of fluorine-containing components, which is consistent with the encapsulation of PHF. According to the TGA curve (Figure 3b), the slight mass loss at 64 °C corresponds to the evaporation of residual surface water, and the prominent weight loss at 120 °C is defined as the thermal triggering temperature of PHF MCs. The encapsulation efficiency of PHF MCs is calculated to be 87.78%, which is superior to most similar thermoresponsive microcapsules. According to TGA curve, the first mass-loss peak appeared at 50 °C, probably because a small amount of PHF was wrapped in the shallow layer of MCs. The significant mass loss around 120 °C is defined as the thermal trigger temperature of PHF MCs, and the packaging efficiency is 87.78%, which is higher than other thermal response MCs [6,35]. After heating at 60 °C for 24 h, the mass loss of PHF MCs is only 5.88%, which is attributed to slight PHF leakage via tiny defects on MUF shell caused by internal vapor pressure. After heating at 60 °C for 24 h, the mass loss of the microcapsules was 5.88% (Figure 3c); after sealed storage at room temperature for 360 days, the mass loss was 1.8% (Figure 3d). These results indicate that the PHF MCs possess a certain degree of encapsulation efficiency and storage stability.

### 3.3. Structure and Chemical Composition Analysis of PHF MCs

**Figure 4:**
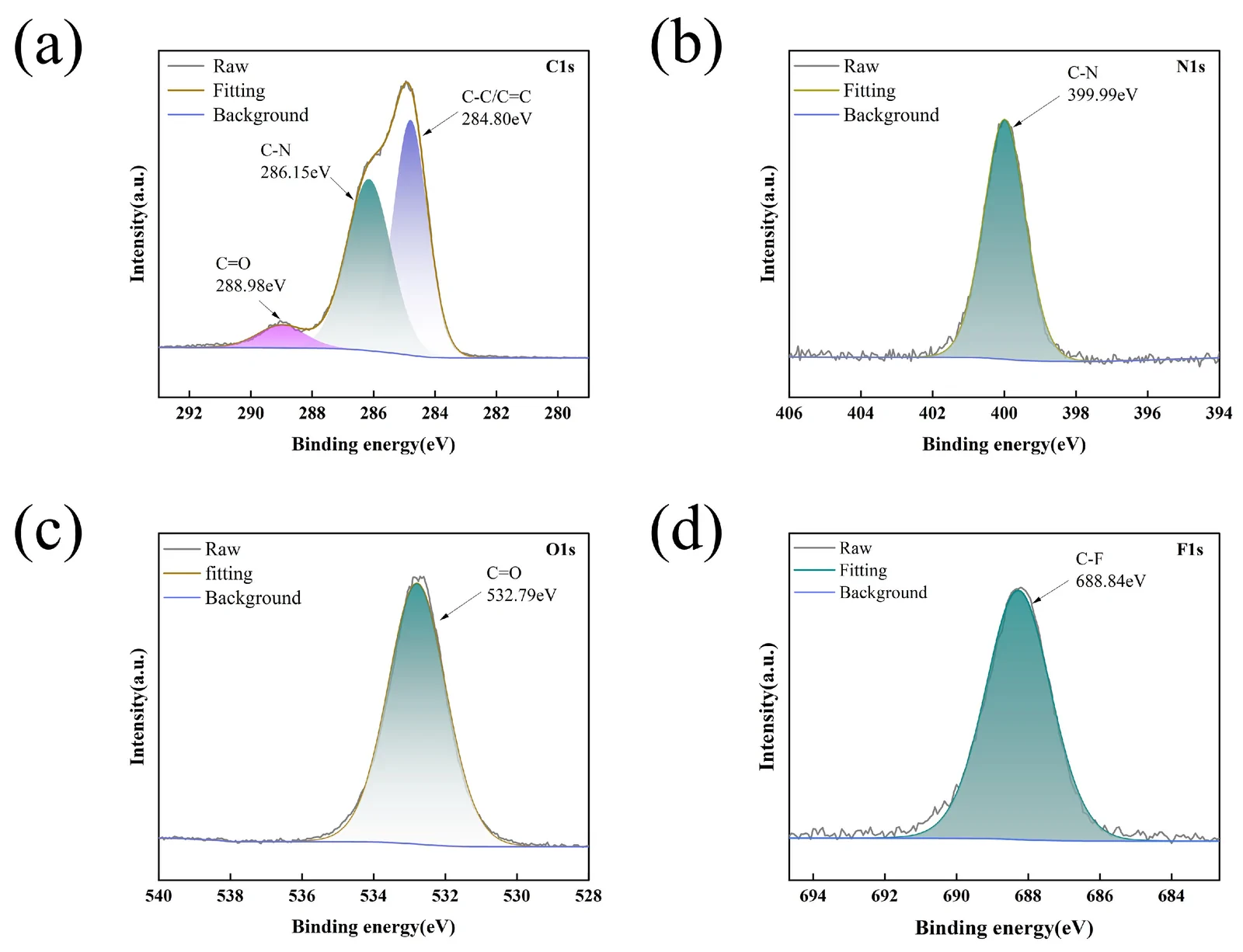
The XPS analysis of PHF MCs. The binding energy of (**a**) C 1s, (**b**) N 1s, (**c**) O 1s, and (**d**) F 1s.

XPS was further employed to analyze the surface elemental composition of the PHF MCs, and the results are presented in Figure 4. The high-resolution XPS spectra revealed the presence of C, N, O, and F elements on the sample surface. In the C 1s spectrum, the peaks near 284.80 eV, 286.15 eV, and 288.98 eV can be assigned to C–C/C–H, C–N, and C=O chemical environments, respectively. The peak near 399.99 eV in the N 1s spectrum can be attributed to C–N structures [36], and the peak near 532.79 eV in the O 1s spectrum is associated with C=O or C–O bonds. The peak near 688.84 eV in the F 1s spectrum indicates the presence of C–F bonds on the sample surface [37].

### 3.4. Morphology Analysis of Composite Materials

**Figure 5:**
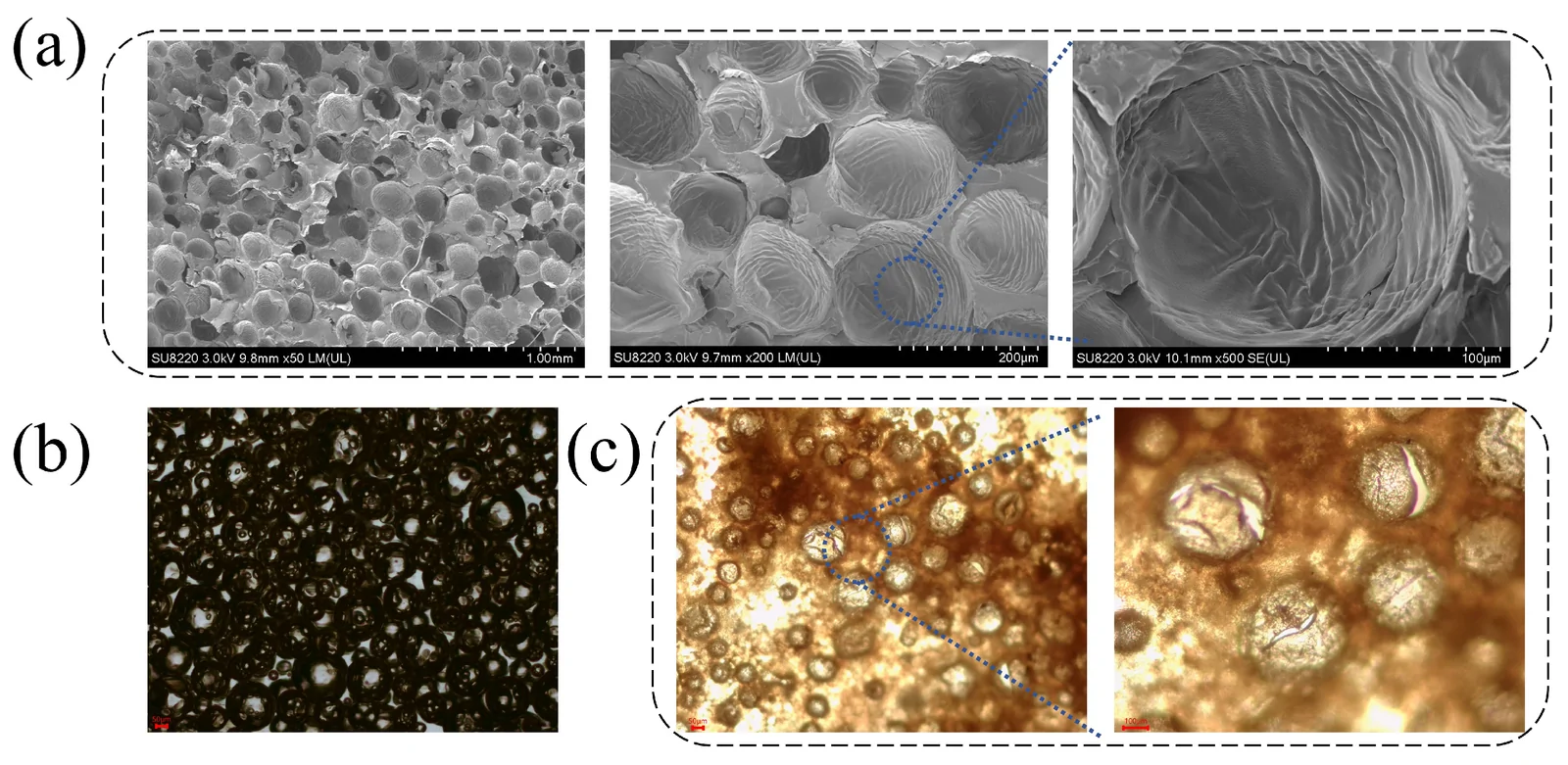
Morphological characterization of the PHF MCs/epoxy resin composite: (**a**) cross-sectional SEM morphology of the composite at different magnifications; (**b**) optical microscopy morphology of the as-prepared composite; (**c**) optical microscopy morphology of the composite after triggered release.

To improve the handling convenience of PHF MCs and construct sheet-type active fire-extinguishing materials, the PHF MCs were incorporated into an epoxy resin matrix and cured at room temperature to prepare sheet-like specimens. Figure 5a shows the cross-sectional morphology with 40% PHF MCs content as a representative proportion; The particles in the sample with low filling amount (10–30%) are sparsely distributed, while the particle density in the sample with high filling amount (50–70%) increases but there is still no obvious agglomeration, and the dispersion uniformity does not deteriorate substantially with the increase of content. The interface between particles and epoxy matrix is closely combined, and no obvious gap or debonding is found, but local particles can be observed in contact with particles at high loading. Cross-sectional SEM images showed that the PHF MCs were relatively uniformly dispersed in the epoxy matrix without obvious large-scale agglomeration. After room-temperature curing, some microcapsules still maintained their spherical structure, indicating a certain degree of structural stability during the mixing and curing processes. Optical microscopy (Figure 5b) results indicated that the majority of the microcapsules remained intact in the as-prepared specimens. After triggered release (Figure 5c), partial rupture or morphological changes of some microcapsules could be observed, suggesting that the core release process may have occurred.

### 3.5. Comprehensive Performance Characterization of Composites

**Figure 6:**
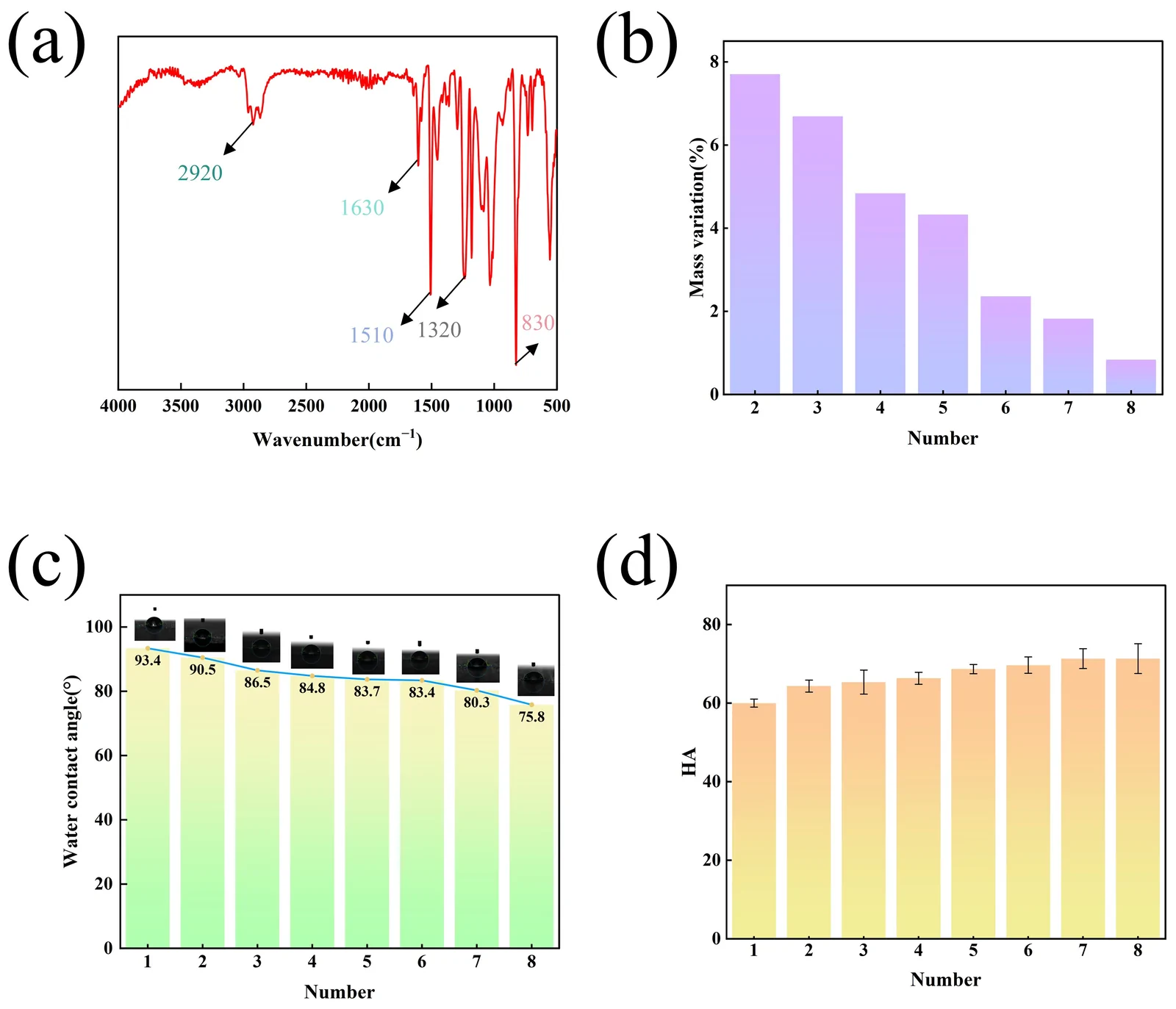
Comprehensive performance characterization of composites: (**a**) FTIR spectra; (**b**) mass loss rate after fire suppression test; (**c**) water contact angle; (**d**) Shore A hardness.

The structural and comprehensive properties of the composite were characterized, and the results are shown in Figure 6. In the FTIR spectrum, the absorption peaks near 2920, 1630, 1510, 1320, and 830 cm^−1^ can be assigned to alkyl C–H stretching vibrations, C=O/amide-related vibrations of the MUF shell, aromatic ring skeleton vibrations of the epoxy resin, C–F stretching vibrations, and out-of-plane bending vibrations of substituted aromatic C–H bonds, respectively. The composite system simultaneously retained the characteristic peaks of the epoxy matrix, the MUF shell, and PHF, and no distinct new peaks were observed, indicating that the three components are merely physically blended. The microcapsules maintained their chemical structural stability during the composite preparation and curing processes and did not react chemically with the matrix.

As the microcapsule content increased, the mass loss rate of the composite after a single fire-extinguishing test decreased from 7.6% to 0.8%, and the surface water contact angle decreased from 93.4° to 75.8°, indicating a reduction in surface hydrophobicity. Meanwhile, the Shore A hardness of the composite increased from approximately 60 HA to about 71 HA. This trend suggests that a high PHF MC content promotes thermally triggered release and mass loss, while the introduction of microcapsules as a dispersed phase may increase the load-bearing capacity of the continuous cured epoxy phase, thereby increasing the hardness. These results indicate that the microcapsule content needs to be comprehensively balanced among fire-extinguishing efficiency, mechanical properties, and surface properties.

From an application perspective, the patch should be regarded as an auxiliary means for local incipient-fire suppression rather than a standalone fire-extinguishing system. It should be applied to surfaces that may be reached by a potential fire plume, and its coverage area should encompass the fire-projection area with an appropriate margin. More importantly, the practical dosage should be specified in terms of the effective areal PHF loading (g/m^2^) rather than solely by the microcapsule mass fraction. The quantitative relationship between this areal loading and the extinguishing time, as well as the triggering reliability after aging, still needs to be determined through scenario-based small-scale fire tests. Considering the self-extinguishing time and hardness variation, a microcapsule content of 40–60% may represent a compromise between fire-extinguishing efficiency and matrix performance; however, the corresponding tensile load-bearing capacity requires further experimental confirmation.

**Figure 7:**
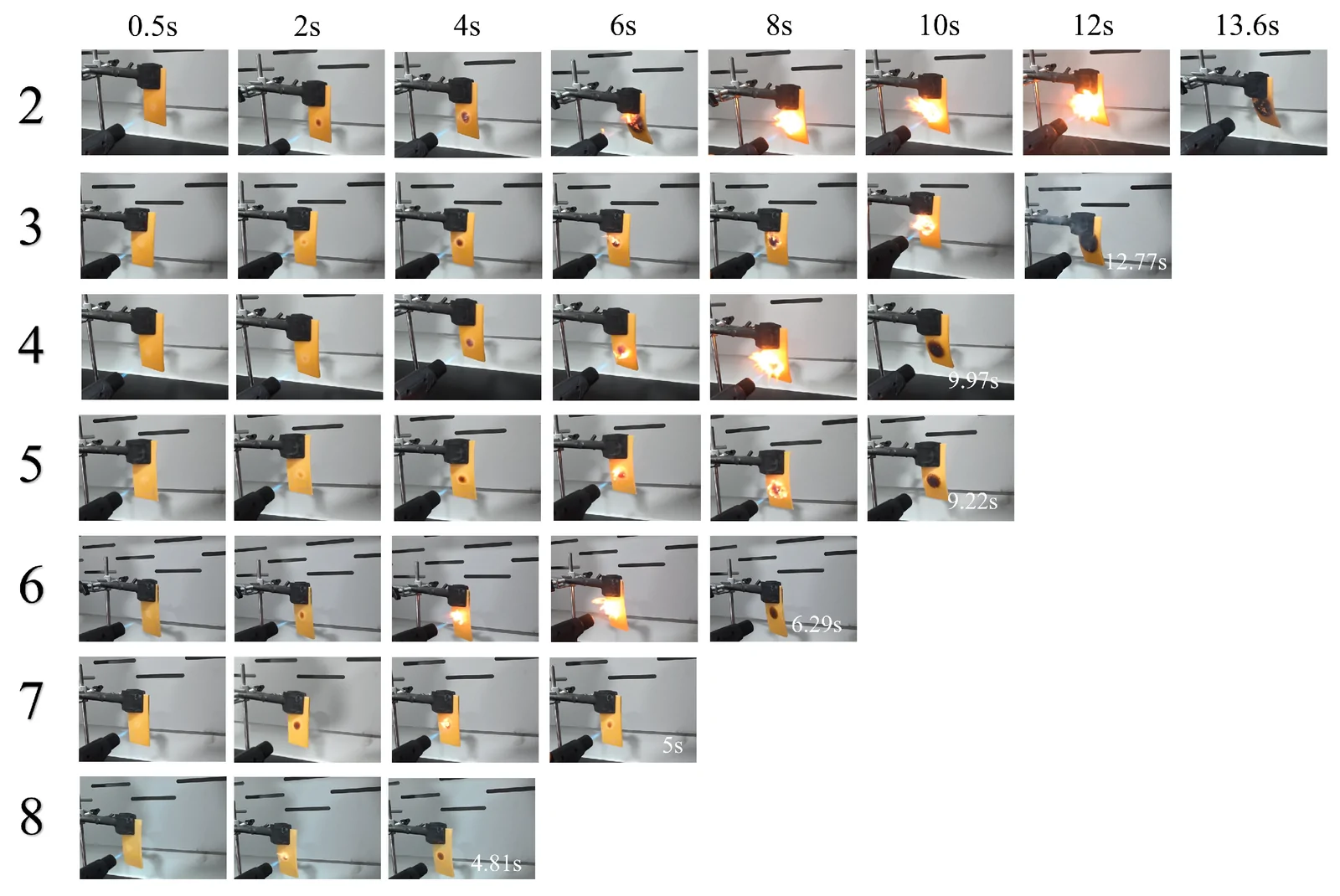
Combustion test of the PHF MCs/epoxy resin composite.

Figure 7 presents the flame combustion behavior of the composite patches with different PHF MC loadings. As the microcapsule content increased from sample 2 to sample 8, the self-extinguishing time decreased from 13.6 s to 4.81 s, indicating that the incorporation of PHF MCs contributed to enhancing the flame suppression capability of the composites. The minimum fire-extinguishing time of gelatin-based PHF microcapsules such as Li [6] is 15 s, and polyurethane interfacial polymerization PHF microcapsules such as Liu [16] can be completely extinguished within 40 s in simulated switchgear. Some samples exhibited a brief flame intensification before extinction, which may be related to gas disturbance caused by the concentrated rupture of microcapsules, the release of local combustible pyrolysis products, or the fact that the concentration of the fire-extinguishing agent had not yet reached the effective suppression threshold. Samples with high loadings could release more PHF at an early stage upon heating, which was beneficial for reducing the radical concentration in the flame zone and suppressing flame spread; samples with low loadings, due to the limited reserve of the fire-extinguishing agent, displayed longer self-extinguishing times and more pronounced ablation.

In order to systematically evaluate the flame retardancy of PHF MCs/epoxy composite patch, UL-94 vertical combustion test was used to investigate its combustion behavior under flame exposure, and the results are shown in Table 2. The residual flame time of the pure epoxy control sample was more than 50 s after two times of flame application, and molten drops appeared to ignite absorbent cotton, which did not reach UL-94 rating. After the introduction of PHF MCs, the afterflame time of the composite patch was significantly shortened, and no dripping ignited absorbent cotton was observed. t_1_ and t_2_ of samples 2 and 3 decreased to 13s, reaching V-1 level; the second afterflame time of sample 4 is slightly higher than 10 s, which is still V-1. When the mass fraction of PHF MCs increased to 40–70%, both t_1_ and t_2_ decreased below 10 s, and there was no dripping ignition, reaching V-0 level. This trend is consistent with the result that the self-extinguishing time is shortened with the increase of microcapsule content in Figure 7.

**Figure 8:**
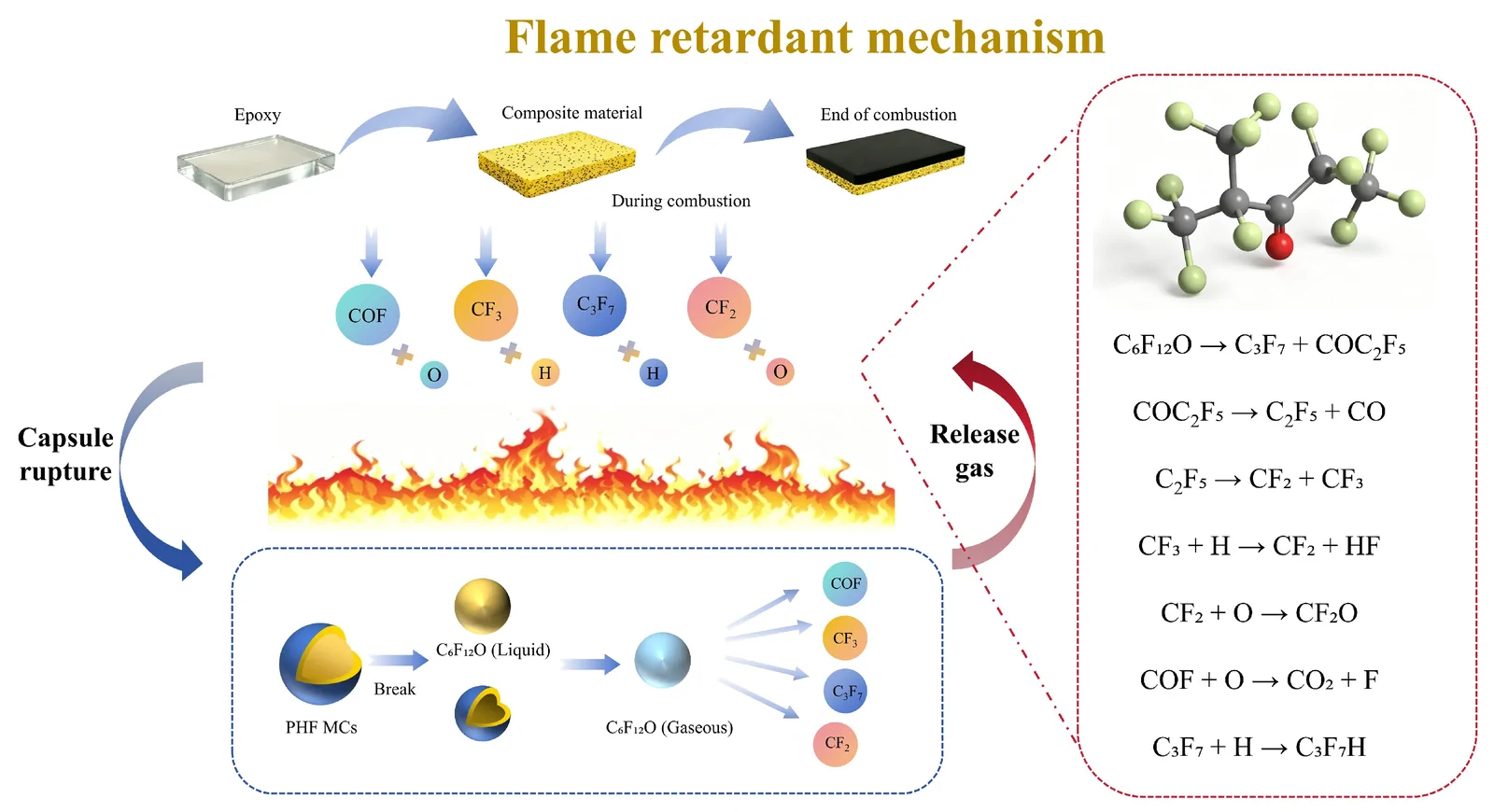
Flame-retardant mechanism of PHF MCs/epoxy composites.

Figure 8 presents a schematic illustration of the flame-retardant and fire-extinguishing mechanism of the PHF MCs/epoxy resin composite. At room temperature, the PHF MCs are embedded as a dispersed phase within the epoxy matrix, and the MUF shell serves to reduce the volatilization and migration of liquid perfluorohexanone under ambient conditions.

Upon heating, PHF vaporizes and is released into the flame zone through shell rupture, increased permeability, or local defects. The released perfluorohexanone reduces the intensity of the combustion reaction primarily through endothermic vaporization, dilution of combustible gases, and gas-phase radical inhibition. The encapsulated PHF vaporizes and undergoes thermal decomposition, generating two primary radicals, C_3_F_7_ and COC_2_F_5_ [16]. Among them, COC_2_F_5_ eliminates CO to form C_2_F_5_, which further decomposes into small reactive species such as CF_2_ and CF_3_, thereby achieving the stepwise release of flame-retardant active centers [38]. CF_3_ can combine with the key combustion chain carrier H to form stable HF, interrupting the propagation pathway of the hydrogen–oxygen combustion chain. CF_2_ reacts with O in the system to generate CF_2_O, consuming the reactive centers of the oxidation reaction. Meanwhile, intermediates such as COF and C_3_F_7_ also synergistically scavenge O and H radicals [37]. In the condensed phase, the epoxy resin may form a char residue or a pyrolytic residual layer upon heating, which exerts a certain barrier effect on heat and oxygen transfer.

## 4. Conclusions

Focusing on the stable loading and thermal trigger release of volatile perfluorohexanone in solid resin matrix, the core–shell microcapsules of MUF coated with PHF were constructed, which were cured with epoxy resin to form a patch material with thermal response and active fire-extinguishing ability. The results show that in situ polymerization of MUF shell is a feasible way to realize the stable loading of volatile PHF in epoxy matrix: microcapsules maintain a relatively complete core–shell structure during the process of epoxy mixing and room temperature curing, and there is no large-scale fracture or chemical structure change.

After PHF MCs were compounded with epoxy resin, the regulation mechanism of microcapsule filling amount on flame-retardant behavior and mechanical properties of the material was systematically revealed. It is confirmed that PHF microcapsules can be uniformly dispersed in epoxy matrix, and will not cause serious interfacial debonding within a suitable filling range. Under the heating condition, microcapsules can directionally release perfluorohexanone gas-phase fire-extinguishing agent, inhibit combustion through gas-phase quenching free radical mechanism, and greatly reduce the ablation mass loss of epoxy matrix.

This work proves that the introduction of perfluorohexanone microcapsules into epoxy matrix is a feasible intrinsic self-extinguishing modification strategy, and the composite material can release the extinguishing agent automatically without additional external supply when triggered by fire, which provides a new idea for the development of epoxy resin disaster prevention functional materials.

## Figures and Tables

**Table 1:** Formulations of epoxy-based fire suppression composites.

Number	Epoxy (g)	Solidifying Agent (g)	PHF MCs (g)	PHF MCs Proportion
1	80	20	0	0
2	80	20	11.12	10%
3	80	20	25	20%
4	80	20	42.86	30%
5	80	20	66.67	40%
6	80	20	100.00	50%
7	80	20	150.00	60%
8	80	20	233.33	70%

**Table 2:** UL-94 vertical combustion test results of pure epoxy control and PHF MCs/epoxy composite patch.

Sample Number	PHF MCs Mass Fraction/%	t_1_/s	t_2_/s	Whether Dripping Ignited Absorbent Cotton	UL-94 Rating
1	0	>50	>50	yes	no
2	10	13.6	13.8	no	V-1
3	20	12.7	12.4	no	V-1
4	30	10.0	10.3	no	V-0
5	40	9.3	9.2	no	V-0
6	50	6.3	6.5	no	V-0
7	60	4.9	5.0	no	V-0
8	70	4.8	4.7	no	V-0

## Data Availability

The original contributions presented in this study are included in the article. Further inquiries can be directed to the corresponding author.

## References

[1] Pan Y., Liu L., Zhang Y., Song L., Hu Y., Jiang S., Zhao H. (2019). Effect of genipin crosslinked layer-by-layer self-assembled coating on the thermal stability, flammability and wash durability of cotton fabric. Carbohydr. Polym..

[2] Cho W., Shields J.R., Dubrulle L., Wakeman K., Bhattarai A., Zammarano M., Fox D.M. (2022). Ion—Complexed chitosan formulations as effective fire-retardant coatings for wood substrates. Polym. Degrad. Stab..

[3] Shi Q., Qin B., Xu Y., Hao M., Shao X., Zhuo H. (2022). Experimental investigation of the drainage characteristic and stability mechanism of gel-stabilized foam used to extinguish coal fire. Fuel.

[4] Burkholder J.B., Cox R.A., Ravishankara A.R. (2015). Atmospheric Degradation of Ozone Depleting Substances, Their Substitutes, and Related Species. Chem. Rev..

[5] Wen C., Meng X., Huber M.L., Wu J. (2017). Measurement and Correlation of the Viscosity of 1,1,1,2,2,4,5,5,5-Nonafluoro-4-(trifluoromethyl)-3-pentanone. J. Chem. Eng. Data.

[6] Li J., Xing Y., Liu H., Lu L., Xue T., Xu X., Zhang F. (2025). Fabrication of thermally activated fire-extinguishing microcapsules and silicone rubber composite. Compos. Commun..

[7] He S., Wang R., Zuo W., Sheng H., Cao C., Huang Y. (2026). Perfluorohexanone & water gel encapsulated in hydrophobic silica aerogel for fire suppression. Powder Technol..

[8] Han Z., Li Z., Yang J. (2025). Highly effective suppression of accidental combustion and explosion on B/KNO3 by perfluorohexanone microcapsule. Fuel.

[9] Pan Q., Sang N., Zhou T., Wu C., Si T., Huang F., Zhu Z. (2025). Array-structured microcapsule fibers for efficient fire extinguishing in confined spaces. Lab A Chip.

[10] Liu Y., Liu J., Zhao Z., Ma Y., Duan Q., Li H., Sun J., Wang Q. (2024). The efficiency of dodecafluoro-2-methylpentan-3-one in suppressing NCM 811 lithium-ion battery fire. Process Saf. Environ. Prot..

[11] Wang Z., He C., Geng Z., Li G., Zhang Y., Shi X., Yao B. (2024). Experimental study of thermal runaway propagation suppression of lithium-ion battery module in electric vehicle power packs. Process Saf. Environ. Prot..

[12] Liu F., Bai Z., Zhang P., Liu Y., Chen B., Gao J. (2026). Preparation and release dynamics of perfluorohexanone thermosensitive microcapsules. J. Therm. Anal. Calorim..

[13] Bai X., Wei R., Huang Q., Li G., Chen Q., Long Y., Xie J., Ding H., Wang C., Zhou T. (2026). Construction of gelatin bio-based polymer microcapsules for encapsulation of perfluorohexanone fire extinguishers. Appl. Mater. Today.

[14] Bai J., Tan Y., Wang K., Zhu C., Yu X., Zong R. (2026). One-step generation and characterization of perfluorohexanone emulsions via modified Tessari method for sodium alginate-based microcapsule particles. Carbohydr. Polym..

[15] Li C., Bian H., Ding D., Huang F., Zhu Z. (2024). Enhancing safety in small confined spaces with thermally triggered fire-extinguishing microcapsules from microfluidics. Lab A Chip.

[16] Liu Z., Li Y., Li Q., Fei B., Pan R., Zhou X. (2026). Water-resistant and flame-retardant waterborne polyurethane coating based on hydrophobic SiO2 and microcapsule perfluorohexanone: With automated intelligent fire early warning and fire protection. Prog. Org. Coat..

[17] Bai X., Wei R., Huang Q., Li G., Chen Q., Long Y., Xie J., Ding H., Wang C., Zhou T. (2026). Expandable microcapsules for enhancing the fire safety of battery pack foam sealants. Chem. Eng. J..

[18] Han Y., Du W., Zhang Y., Qiu C., Law M., Zhao Y., Tang B.Z., Wang Y., Yang J. (2026). Programming Interfacial Polymerization: Machine Learning Unveils Quantitative Rational Design Rules for Microcapsules and Beyond. Adv. Mater..

[19] Hu D., Zhang B., Zhang Z., Huang F., Zhang G., Zhang G., Ma C. (2026). In Situ Fabricating Rigid Polyurethane Foams With Lightweight yet High-Strength, Passive Flame-Retardant, and Active Fire-Fighting Properties. Adv. Funct. Mater..

[20] Ren Y., Zhao Z., Wang C., Li C., Zhang J., Zhang P., Fu J., Huang F., Zhu Z. (2026). Rapid cooling of lithium-ion battery thermal runaway achieved by high-silica fabric based on dual-functional fire-extinguishing microcapsule coating. Chem. Eng. J..

[21] Guo X., Lu C., Li Y., Zhang C., Pan X., Hua M., Zhang L., Jiang J. (2023). Preparation and fire extinguishing mechanism of core-shell solid-liquid composite powder containing ammonium dihydrogen phosphate. Powder Technol..

[22] Ni X., Zheng Z. (2020). Extinguishment of sodium fires with Graphite@Stearate core-shell structured particles. Fire Saf. J..

[23] Hu Z.-T., Reinack V.H., An J., Indraneel Z., Dasari A., Yang J., Yang E.-H. (2021). Ecofriendly Microencapsulated Phase-Change Materials with Hybrid Core Materials for Thermal Energy Storage and Flame Retardancy. Langmuir.

[24] Tianwei Z., Cunwei Z., Hao L., Zhiyue H. (2020). Experimental investigation of novel dry liquids with aqueous potassium Solution@Nano-SiO2 for the suppression of liquid fuel fires: Preparation, application, and stability. Fire Saf. J..

[25] Yim T., Park M.-S., Woo S.-G., Kwon H.-K., Yoo J.-K., Jung Y.S., Kim K.J., Yu J.-S., Kim Y.-J. (2015). Self-Extinguishing Lithium Ion Batteries Based on Internally Embedded Fire-Extinguishing Microcapsules with Temperature-Responsiveness. Nano Lett..

[26] Yang Y., Xu Y., Ji Y., Wei Y. (2021). Functional epoxy vitrimers and composites. Prog. Mater. Sci..

[27] Jin F., Li X., Park S. (2015). Synthesis and application of epoxy resins: A review. J. Ind. Eng. Chem..

[28] Sangermano M., Bergoglio M., Schögl S. (2024). Biobased Vitrimeric Epoxy Networks. Macromol. Mater. Eng..

[29] Tang Y., Zhang X., Liu X., Wei W., Li X. (2024). Facile synthesis of hyperbranched epoxy resin for combined flame retarding and toughening modification of epoxy thermosets. Chem. Eng. J..

[30] Ma C., Qiu S., Wang J., Sheng H., Zhang Y., Hu W., Hu Y. (2018). Facile synthesis of a novel hyperbranched poly(urethane-phosphine oxide) as an effective modifier for epoxy resin. Polym. Degrad. Stab..

[31] Ratna D., Becker O., Krishnamurthy R., Simon G.P., Varley R.J. (2003). Nanocomposites based on a combination of epoxy resin, hyperbranched epoxy and a layered silicate. Polymer.

[32] Toldy A., Anna P., Csontos I., Szabó A., Marosi G. (2007). Intrinsically flame retardant epoxy resin—Fire performance and background—Part I. Polym. Degrad. Stab..

[33] Mathews L.D., Capricho J.C., Salim N., Parameswaranpillai J., Moinuddin K., Hameed N. (2023). Intrinsically modified self-extinguishing fire-retardant epoxy resin using boron-polyol complex. J. Polym. Res..

[34] Li S., Chen D., Gao C., Yuan Y., Wang H., Liu X., Hu B., Ma J., Liu M., Wu Z. (2020). Epoxy-functionalized polysiloxane/Nano-SiO2 synergistic reinforcement in cryogenic mechanical properties of epoxy and carbon fiber reinforced epoxy laminate. Compos. Sci. Technol..

[35] Liu H., Zhang T., Zhang M., Zhang C., Guo Z., Zhang Y., Chen H., Wu Y., Zhang G. (2024). Preparation and thermal responsiveness of microencapsulated fluorinated liquids for automatic fire extinguishing. Heliyon.

[36] Liu Q., Luo H., Gao Z., Huang Y., Liang J., Zhou H., Sun J. (2024). Preparation of waterborne intumescent flame-retardant coatings using adenosine-based phosphonates for wood surfaces. Prog. Org. Coat..

[37] Tian S., Yao C., Huang Z., Maluta N.E., Yang M., Zhang X. (2026). In-situ polymerized B4C-MUF@perfluoro(2-methyl-3-pentanone) microcapsules for enhanced thermal stability and synergistic flame retardancy. Polym. Degrad. Stab..

[38] Zhang J., Gao W., Chen L., Li Y. (2024). Inhibition and enhancement of hydrogen explosion by perfluorohexanone. Int. J. Hydrogen Energy.

